# Human monocarboxylate transporters accept and relay protons via the bound substrate for selectivity and activity at physiological pH

**DOI:** 10.1093/pnasnexus/pgad007

**Published:** 2023-01-18

**Authors:** Katharina Geistlinger, Jana D R Schmidt, Eric Beitz

**Affiliations:** Department of Pharmaceutical and Medicinal Chemistry, Christian-Albrechts-University of Kiel, Gutenbergstraße 76, Kiel 24118, Germany; Department of Pharmaceutical and Medicinal Chemistry, Christian-Albrechts-University of Kiel, Gutenbergstraße 76, Kiel 24118, Germany; Department of Pharmaceutical and Medicinal Chemistry, Christian-Albrechts-University of Kiel, Gutenbergstraße 76, Kiel 24118, Germany

**Keywords:** monocarboxylate transporter, lactate, proton, mechanism, mutagenesis

## Abstract

Human monocarboxylate/H^+^ transporters, MCT, facilitate the transmembrane translocation of vital weak acid metabolites, mainly l-lactate. Tumors exhibiting a Warburg effect rely on MCT activity for l-lactate release. Recently, high-resolution MCT structures revealed binding sites for anticancer drug candidates and the substrate. Three charged residues, Lys 38, Asp 309, and Arg 313 (MCT1 numbering) are essential for substrate binding and initiation of the alternating access conformational change. However, the mechanism by which the proton cosubstrate binds and traverses MCTs remained elusive. Here, we report that substitution of Lys 38 by neutral residues maintained MCT functionality in principle, yet required strongly acidic pH conditions for wildtype-like transport velocity. We determined pH-dependent biophysical transport properties, Michaelis–Menten kinetics, and heavy water effects for MCT1 wildtype and Lys 38 mutants. Our experimental data provide evidence for the bound substrate itself to accept and shuttle a proton from Lys 38 to Asp 309 initiating transport. We have shown before that substrate protonation is a pivotal step in the mechanisms of other MCT-unrelated weak acid translocating proteins. In connection with this study, we conclude that utilization of the proton binding and transfer capabilities of the transporter-bound substrate is probably a universal theme for weak acid anion/H^+^ cotransport.

**Significance Statement**

l-Lactate is a monocarboxylate anion derived from deprotonation of l-lactic acid. Cells need to release l-lactate and the accompanying proton to maintain their energy metabolism. Secondary-active monocarboxylate/proton cotransporters, MCT, carry the main load of human l-lactate transport. MCT inhibitors are currently developed as anticancer drugs. Cryo-EM structures revealed binding sites for l-lactate and inhibitors. However, a fundamental question remained, namely how the cotransported proton binds to and traverses the transporter. We elucidated the proton transfer mechanism by comparing transport properties of MCT wildtype with point mutants. This way, we found that the bound l-lactate itself represents the missing proton binding and transfer site. Further examples of MCT-unrelated proteins suggest that substrate protonation is a universal principle in weak acid transport.

## Introduction

Metabolic monocarboxylic acids largely deprotonate in the human body due to an acidic pK_a_ around 4 (1). The resulting monocarboxylates represent typically more than 99% of the equilibrium species at physiological pH (2). Transmembrane diffusion of the anions is strongly impeded and dedicated transporters are required for release as metabolic waste or uptake as nutrients. Transport of the monocarboxylate alone, however, would have unfavorable consequences in a physiological setting. First, anion transport is electrogenic. While the negative membrane potential would drive the export of monocarboxylates, import would be strongly hindered (3). This would basically prohibit the use of monocarboxylates as nutrients. Second, monocarboxylates, e.g. pyruvate or l-lactate, that derive from metabolic redox processes such as glycolysis are produced together with one proton each. Export of the monocarboxylate alone would result in detrimental acidification of the cytosol by the remaining protons if not resolved by energy-consuming H ^+^ -ATPase activity (4).

Four monocarboxylate transporters, MCT1-4, of the SLC16 family carry the main load of human transmembrane l-lactate transport by a proton cotransport mechanism (3–6). Their physiological role is to maintain energy metabolism. MCTs are further at the core of the Warburg effect of glycolytic tumors that is characterized by the production of large amounts of l-lactate despite an adequate supply of oxygen (7). Circulating l-lactate can fuel oxidative tumor cells via MCTs (8). Therefore, MCT inhibitors are being developed as anticancer drugs (9).

**Fig. 1.**
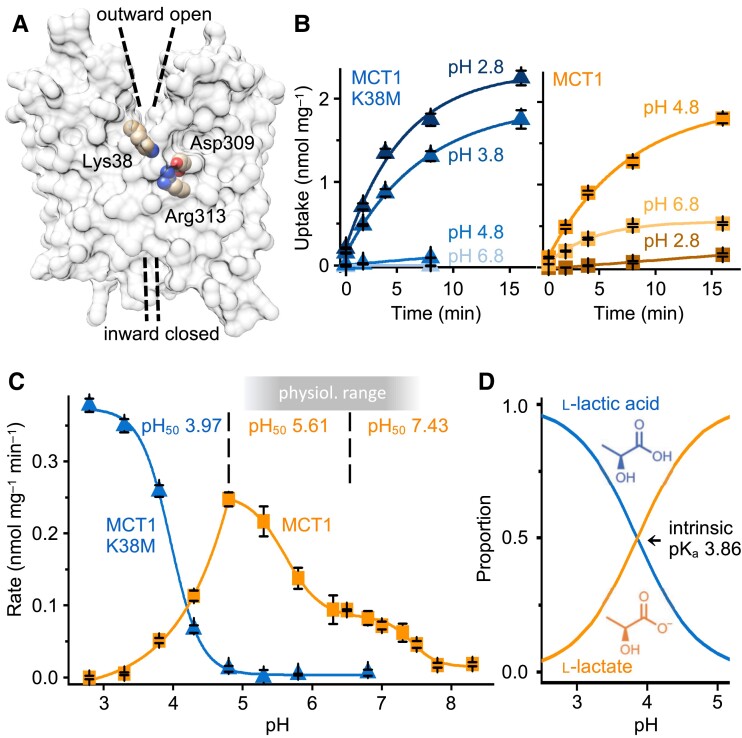
Transport functionality of MCT1 K38 mutants at acidic pH. (A) Structure of human MCT1 in the outward open conformation (PDB# 6LZ0). The charged amino acid residues of the substrate binding site are shown as spheres. (B) l-Lactate uptake over time into yeast via MCT1 K38M (left, blue curves), and MCT1 wildtype (right, orange) at a 1 mM inward substrate gradient and different buffer pH conditions. Error bars denote SEM (n = 3). (C) Dependence of the l-lactate uptake rate on pH of MCT1 K38M (blue curve) and MCT1 wildtype (orange curve). Error bars denote SEM (n = 3–6). (D) Protonation equilibrium of l-lactate/l-lactic acid. The orange line depicts the proportion of the l-lactate species at a given buffer pH, the neutral l-lactic acid species is shown in blue.

Recently, high-resolution MCT structures were generated to elucidate the molecular transport mechanism and drug candidate binding sites (10–12). The overall MCT protein structure comprises 12 transmembrane helices with an internal symmetry of the first and second helix bundle probably originating from gene duplication (13). MCTs undergo a large conformational change during transport by rigid-body rotation supporting an alternating access transport mechanism. Drug-like molecules lock MCT1 in its outward- or inward-open conformation, and a D309N mutation fixed MCT1 in the inward-open state in the absence of an inhibitor (12). In wildtype MCT1, Asp 309 forms a salt bridge with Arg 313 (Fig. 1A) (14). Breakage of the interaction by protonation of Asp 309 has been hypothesized to initiate the alternating access transition (12). A third charged amino acid residue, Lys 38, is present at the MCT1 substrate binding site. Lys 38 with a protonated ɛ-amine was suspected before to represent a major binding site for the monocarboxylate substrate (15). Mutation of either of the three charged residues reportedly rendered MCT1 nonfunctional (10, 12, 15, 16). While the previous functional and structural studies identified essential residues for substrate binding and the transport process, the proton binding site and cotransport mechanism remained elusive or speculative.

Here, we expressed human MCT1 and Lys 38/Asp 309/Arg 313 mutation variants in a *Saccharomyces cerevisiae* strain lacking endogenous l-lactate transporting proteins (17, 18). Contrary to other expression systems, background transport is marginal in this strain, and the absence of l-lactate metabolizing enzymes allows for prolonged assay times to reach the transport equilibrium state (17). Key to this study, yeast tolerates strongly acidic external pH conditions. When we assayed a MCT1 K38M mutant at pH 3.8 and pH 2.8, it unexpectedly exhibited transport velocities above wildtype MCT1 at its pH 4.8 optimum. Starting out from this result, we related pH-dependent biophysical transport properties, Michaelis–Menten kinetics, and heavy water effects of MCT1 wildtype to mutation variants of Lys 38. This revealed the proton cotransport mechanism in which the bound substrate shuttles a proton from Lys 38 to Asp 309 initiating the alternating access conformational change. The required proton transfer across the substrate is a key element in the selectivity mechanism of the MCT family of monocarboxylate transporters.

## Results

We started out by generating point mutants of human MCT1 in which the charged residues of the l-lactate binding site were individually replaced by amino acids with neutral sidechains, i.e. MCT1 K38M, MCT1 D309N, and MCT1 R313N. The Arg 313 position was mutated to asparagine because this exchange is naturally present in another human MCT, i.e. MCT12 a transporter for zwitterionic creatine. We expressed the MCT1 mutants in *S. cerevisiae* W303-1A Δ*jen1* Δ*ady2* lacking endogenous monocarboxylate transporters (Figs. S1A and S1B).

### MCT1 Lys 38 mutants are functional at acidic pH

Initially, we assayed uptake of ^14^C-l-lactate in the absence of a transmembrane proton gradient at pH 6.8 (19). As reported by others (10, 12, 15, 16), at these conditions, MCT1 mutants with altered Lys 38, Asp 309, or Arg 313 sites appeared nonfunctional (Fig. 1B, Fig. S1C), whereas MCT1 wild-type facilitated l-lactate uptake (Fig. 1B). Transport equilibrium with equal import and export velocities of l-lactate/H^+^ was reached within 16 min. At lower pH, MCT1 D309N and MCT1 R313N remained nonfunctional (Fig. S1C), whereas MCT1 K38M enabled some l-lactate uptake at pH 4.8 (20) and switched to full activity at pH 3.8 and 2.8 (Fig. 1B). MCT1 wildtype was maximally active at pH 4.8, and the inward proton gradient shifted the cytosolic l-lactate concentration to a markedly higher level (Fig. 1B).

We generated more MCT1 Lys 38 mutants, namely MCT1 K38A with a shorter neutral sidechain, MCT1 K38E to reverse the sidechain charge to negative, and MCT1 K38R carrying the two orders of magnitude stronger guanidine base to prevent potential proton release (Figs. S1A and S1B). MCT1 K38A behaved as MCT1 K38M (Fig. S1D). MCT1 K38E was inactive at pH 6.8 and 4.8; yet pH 2.8, which is about one log-unit below the pK_a_ of the glutamate γ-carboxyl, initiated transport (Fig. S1E). This indicates that a glutamate at position 38 is solvent accessible, and partial neutralization by protonation allows for transport similar in fashion to K38M and K38A. MCT1 K38R was nonfunctional at all tested pH conditions (Fig. S1F). Possibly, a permanent charge at position 38 prohibits transport, whereas Lys 38 in MCT1 wildtype may not be charged at all times but could be part of a proton transfer process. It cannot be excluded, though, that the larger size of the arginine sidechain may contribute to the loss of function.

### Lys 38 enables MCT1 transport at physiological pH

Next, we carried out a fine-grained titration of MCT1 wildtype and K38M transport rates against pH to determine the number of accessible protonation sites relevant to l-lactate/H^+^ transport (Fig. 1C). The titration curves appeared strongly shifted, and differed in complexity. The sigmoidal curve of MCT1 K38M indicated a single transport-relevant protonation step. The inflection point of the sigmoidal function (pH_50_) was determined to 3.97 (Fig. 1C, blue curve), which corresponded to the intrinsic pK_a_ of l-lactic acid (pK_a_ 3.86; Fig. 1D). The curve shape and pH_50_ were highly reminiscent of the l-lactic acid permeability of the solute channel aquaporin-9, AQP9 (19, 21) supporting the view that MCT1 K38M equally uses neutral l-lactic acid as a substrate. Accordingly, at sufficiently acidic buffer, the l-lactate anion itself binds and carries the proton cosubstrate for MCT1 K38M.

With a lysine present at position 38 of MCT1 wildtype, l-lactate/H^+^ transport functionality was shifted to neutral and mildly acidic physiological pH (Fig. 1C, orange curve). Further, the curve can be sectioned: a monotonically increasing part depicting an initial raise in the transport rate from neutral pH towards mildly acidic conditions followed by a prominent boost in transport velocity at more acidic conditions. This part was fitted best with a double-sigmoidal function (r^2^ = 0.9978) with inflection points at pH 7.43 and pH 5.61 (Fig. 1C). Transport velocity was maximal at pH 4.8, and exponentially decreased inverse in shape to the pH curve of MCT1 K38M (Fig. 1C). The opposite pH dependence in the most acidic curve section suggests that MCT1 wildtype accepts the l-lactate anion as a substrate rather than neutral l-lactic acid.

A double-sigmoidal curve shape may indicate two buffer-accessible protonation sites that influence the overall transport process. Since this part is absent in the Lys 38-less MCT1 K38M mutant, the protonation sites may be linked to the Lys 38 sidechain ɛ-amine in the substrate-unbound state and to the complex of Lys 38 with l-lactate. While the protonation status of the Lys 38 sidechain appears to be determined by the buffer pH (see also MCT1 K38E mutant; Fig. S1E), we did not notice direct protonation of Asp 309 in our titrations by external protons. This is fostered by solvation simulations based on the protein structure of MCT1 (12) in which bulk-linked water clusters appeared close to Lys 38, whereas water was absent around the Asp 309 site (Fig. S2). Together, the pH data show that MCT1 with replaced Lys 38 is functional in principle; yet, the basicity of the lysine ɛ-amine is required to enable transport at physiological pH.

### Direct evidence for binding of l-lactate to the protonated Lys 38 ɛ-amine

**Fig. 2.**
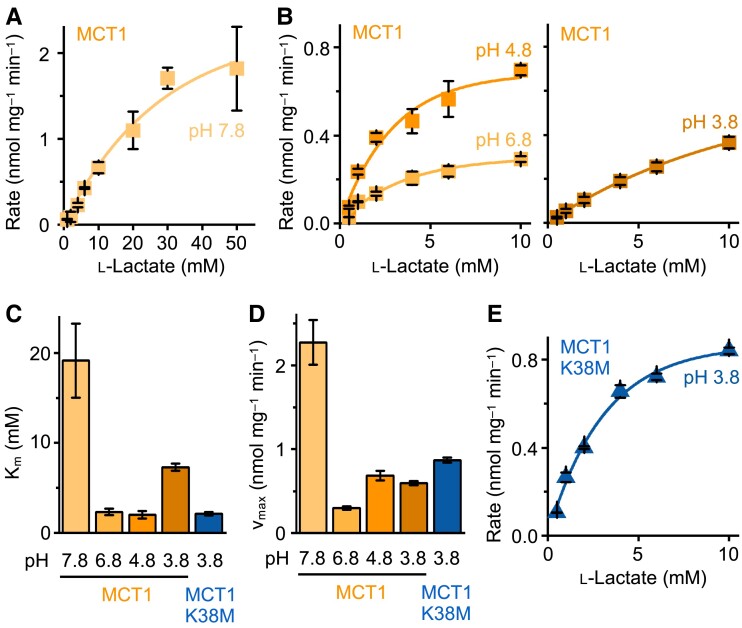
Michaelis-Menten kinetics of l-lactate transport via MCT1 wildtype and MCT1 K38M. (A & B) Rates of l-lactate uptake via MCT1 wildtype at increasing substrate concentrations in a pH range from 7.8 to 3.8 (note the different scaling of the axes in A). (C) Plot of the pH-dependent K_m_ values derived from the curves in A, B, and E. (D) Maximum velocity of transport determined from the measured or extrapolated plateaus of the curves in A, B, and E. (E) Michaelis-Menten curve of l-lactate uptake via MCT1 K38M at pH 3.8. Error bars denote SEM (n = 3).

We figured that if the observed pH_50_ of 7.43 was derived from Lys 38, l-lactate affinity should be strongly decreased at alkaline pH due to deprotonation of the ɛ-amine. Therefore, we determined Michaelis–Menten kinetics (Fig. 2). Indeed, the l-lactate affinity at pH 7.8 appeared one order of magnitude lower (K_m_ 19.2 ± 4.1 mM) than at physiological pH conditions (K_m_ 2.32 ± 0.36 mM) (Figs. 2A–C). The decrease in affinity at pH 3.8 (K_m_ 7.29 ± 0.40 mM) is due to the shift of the l-lactate/lactic acid equilibrium towards the neutral species (Fig. 1D). A pH of 8 was shown to lock MCT1 in the outward-open conformation (12), and a strong decrease in l-lactate affinity at alkaline pH was seen with human erythrocytes in which MCT1 is the prominent l-lactate transporter (22). Together with our data, this provides evidence for a binding mechanism in which a proton binds first to the Lys 38 ɛ-amine and generates a positive charge for the subsequent binding of the l-lactate anion. The observed decrease in affinity at the most acidic buffer pH of 3.8 (Fig. 2C) is due to the shifted l-lactate/lactic acid equilibrium towards the neutral form (Fig. 1D).

The maximum velocity of transport, v_max_, also correlated with the MCT1 Lys 38 protonation status (Fig. 2D). At pH 7.8, i.e. in the mainly uncharged state of Lys 38, transport was up to one order of magnitude faster than at lower pH conditions. We attribute this to less impedance of the substrate at the binding site due to weaker interaction with the neutral Lys 38 sidechain.

To meet the acidic pH requirements of MCT1 K38M, we measured K_m_ at pH 3.8 (Fig. 2E). The substrate affinity (K_m_ 2.13 ± 0.19 mM; Fig. 2C) was equal to MCT1 wildtype at a physiological pH, and v_max_ was higher than that of MCT1 wildtype at pH 6.8 and 4.8, yet not as high as at pH 7.8 (Fig. 2D). An uncharged residue at position 38 is beneficial for transport velocity, and polar interactions with the amine of Lys 38 further accelerate transport. The generally comparable K_m_ and v_max_ values of MCT1 wildtype and K38M suggest that the proton-driven alternating access transport mechanism remained unaltered in principle despite entry of the substrate in different forms, i.e. l-lactate/H^+^ (MCT1 wildtype) vs. l-lactic acid (MCT1 K38M).

### MCT1 is selective for protonatable weak acid anions

**Fig. 3.**
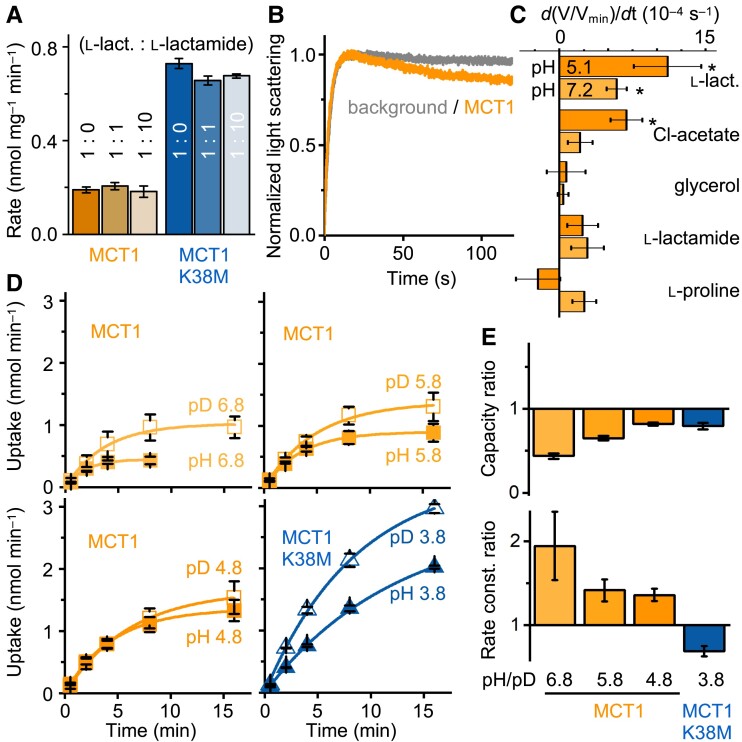
Substrate selectivity of MCT1 wildtype and MCT1 K38M, and heavy water effect. (A) Absence of competition of neutral l-lactamide for transport. Addition of l-lactamide at an equal concentration or 10-fold excess did not inhibit transport of radiolabeled l-lactate into yeast expressing MCT1 wildtype or MCT1 K38M at pH 3.8 (n = 2–3). (B) Stopped-flow light scattering traces showing uptake of l-lactate via MCT1 wildtype (orange) driven by a hyperosmotic inward gradient of 300 mM substrate at pH 5. The initial increase in light scattering (<15 s) is due to cell shrinkage by an osmotic efflux of water. A regain in cell volume by uptake of l-lactate is seen in the second phase (>15 s). Non-expressing cells do not facilitate l-lactate uptake (gray trace) (n = 3). (C) Yeast cell volume change by substrate uptake via MCT1 wildtype measured as stopped-flow light scattering at pH 5.1 and 7.2. Only acidic compounds yielded significant uptake (asterisks, *P* < 0.05, n = 3). (D) Heavy water effect on l-lactate uptake over time into yeast expressing MCT1 wildtype and MCT1 K38M at a 1 mM inward substrate gradient and different buffer pH (closed symbols) and pD conditions (open symbols). (E) Ratio of transport capacities in light and heavy water (top). Ratio of the pH/pD rate constants indicating a normal (slowing) isotope effect on transport via MCT wildtype and a mild inverse (accelerating) isotope effect for MCT1 K38M. Errors bar denote SEM (n = 2).

Next, we tested whether MCT1 wildtype or MCT1 K38M would accept l-lactamide, a permanently neutral mimic of l-lactic acid (Fig. 3A), as a transport substrate. We chose a buffer pH of 3.8 for equal concentrations of l-lactate and l-lactic acid (Fig. 1D). At an equimolar concentration or 10-fold excess of l-lactamide, neither MCT1 wildtype nor MCT1 K38M showed a decrease in the uptake rate by competition (Fig. 3A), indicating that l-lactamide is not an MCT1 or MCT1 K38M substrate.

We employed a second method to test more directly for l-lactamide transport by MCT1 using stopped-flow light scattering (Fig. 3B) (21, 23). This assay requires a steep substrate gradient to elicit sufficiently large changes in cell volume for detection. In the initial phase, the hyperosmotic conditions rapidly drive water out of the yeast eliciting an increase in light scattering (Fig. 3B). The slower import of MCT substrates leads to partial re-swelling and a decrease in light scattering. As in the radiolabeled competition assays, l-lactamide was not transported (Fig. 3C). Testing of further similarly sized compounds fostered the mechanistic findings of this study. We noted that the transport rate of l-lactate and chloroacetate (24) decreased with decreasing pK_a_, i.e. 3.86 and 2.83, respectively. This again indicates that direct substrate protonation is relevant for transport. Neutral glycerol, which is incapable of binding and transferring protons, was excluded from transport by MCT1, as was zwitterionic l-proline (Fig. 3C) (25).

### Bound weak acid substrates shuttle protons from Lys 38 to Asp309

The distance between the Lys 38 ɛ-amine and the Asp 309/Arg 313 salt bridge was determined to about 8 Å (Fig. S2) (12). A substrate carboxyl group appears suited to fill the gap and may shuttle a proton from Lys 38 to Asp 309. Protonation of Asp 309 was suggested to break the salt bridge initiating the alternating access mechanism (12). To gain insight into relevant proton transfer events, we determined the effect of heavy water, D_2_O, on MCT1 transport.

Deuterons have twice the mass of protons, which increases the chemical bond strength resulting in a characteristic pK_a_ shift of monocarboxylic acids by approx. 0.5 (26), and a decrease of the deuteron-hopping velocity (27). The degree by which a proton-dependent chemical process is retarded in heavy water, thus, is indicative of the complexity of the involved transfer chain (28).

We changed the solvent of the yeast suspensions from light water to heavy water at least 30 min prior to the transport assays for adjustment (29, 30). Other than that, the buffers were identically composed and contained the same concentrations of protons or deuterons, i.e. pH or pD. The height of the plateaus derived from the obtained or extrapolated uptake curves (Fig. 3D) indicates the total amount of intracellular substrate in the equilibrium states, termed capacity. Throughout, the capacity of l-lactate/D^+^ transport was higher than with l-lactate/H^+^ (Fig. 3D, Fig. S3A). The heavy water effect was most pronounced at pD 6.8, and higher free deuteron concentrations somewhat compensated the effect (Fig. 3E, top). The rate constants indicated slower transport of MCT1 wildtype in heavy water at all pH/pD conditions (Fig. 3E, bottom, Fig. S3B). MCT1 K38M transport, in turn, appeared slightly accelerated in heavy water. Inverse isotope effects are rare and can indicate that substrate protonation takes place outside of the protein, and other rate limiting events determine the velocity of the overall process (31).

The alternating access transition is usually a major rate-limiting process in the transporter class. Making use of the pre-protonated pool of l-lactic acid in the external buffer may explain the mild inverse isotope effect observed with MCT1 K38M. The normal isotope effect with MCT1 wildtype, in turn, indicates that proton transfers inside the protein are relevant for the overall transport velocity. With the previous observations of this study, this suggests that l-lactate binds to Lys 38 and accepts a proton from the lysine ɛ-amine. The final and common step for MCT1 wildtype and the K38M mutant would be a proton transfer from the substrate to Asp 309 that breaks the salt bridge and initiates the alternative access conformational change.

## Discussion

**Fig. 4.**
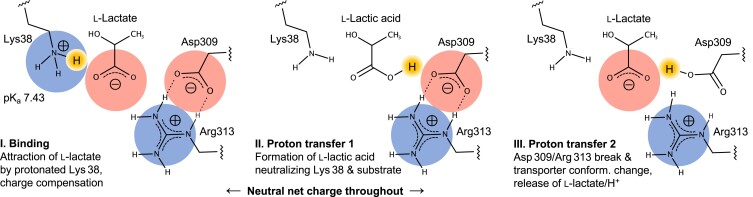
Model of the l-lactate/H^+^ transport mechanism via MCT monocarboxylate transporters. The bound weak acid substrate anion itself acts as a proton acceptor and transfer site establishing selectivity and transport activity in the physiological pH range. See text for details.

The MCT family of monocarboxylate/H^+^ transporters evolved in a way that efficiently enables utilization of the prevalent pool of monocarboxylate anions by exploiting the transmembrane pH gradient generated by the accompanying protons. The data of this study suggest that the bound substrate itself contributes to proton transfer. Since the amino acid residues of the binding site are conserved among the mammalian MCT-type monocarboxylate transporters (Fig. S4), we view the general transport mechanism as follows:

- The substrate anion is electrostatically steered to the MCT binding site by a net charge of plus one originating from the protonated Lys 38 ɛ-amine (Fig. 4, left). The charge compensation results in a neutral protein interior and possibly prevents differently charged organic anions, such as dicarboxylic acids or phosphates, from being accepted as substrates. The principle of transporter charge compensation has been shown before, e.g. for the mitochondrial alternating access ADP/ATP carrier of the SLC25 family (32). ADP has a charge of minus three matching three positively charged residues in the binding pocket for import into the mitochondrial matrix. ATP with a net charge of minus four is transported in the opposite direction driven by the negative membrane potential.
- In a second step, a proton from MCT1 Lys 38 neutralizes l-lactate to form l-lactic acid (Fig. 4, center). This is facilitated by the more lipophilic environment within the protein that leads to convergence of both pK_a_. Only substrates that have the capability to accept and transfer a proton under these conditions can initiate the next step in the transport mechanism. This process would further account for the strict selectivity of MCT1–4 for monocarboxylates.
- Eventually, a proton transfer from l-lactic acid to Asp 309 leads to breakage of the Asp 309/Arg 313 salt-bridge (Fig. 4, right). The MCT protein converts into the inward open state from which l-lactate and the Asp 309-bound proton are released. The transport cycle is complete with the re-protonation of Lys 38. At an effective pK_a_ of 7.43, this should readily occur from the aqueous bulk even at neutral pH conditions.

Generally, water molecules can be part of proton transfer chains. The observed mild heavy water effect, however, is in favor of a transfer chain with a small number of hopping steps. This transport model is compatible with the bidirectionality of MCT transport. In the inward open cryo-EM structure of MCT1 D309N, the Lys 38 ɛ-amine seems accessible to substrate molecules (Fig. S5) (12). Substrate binding from the cytosolic side would reverse the above-described process. Therefore, directionality of transport is determined by the transmembrane substrate and proton gradients via the probability of binding from the extra- and intracellular space. Further, accessory proteins, i.e. the extracellular domain of basigin and isoforms of the carbonic anhydrase family, modulate MCT directionality by exerting antenna functions for substrate anions and/or protons (17, 33).

Currently, three types of membrane proteins have been functionally and structurally described that facilitate transmembrane translocation of a weak acid anion plus a proton. These are members of the ubiquitous AQP family (19, 21, 34, 35), microbial formate/nitrate transporters, FNT (2, 36–38), and MCT-type monocarboxylate transporters. All use electrostatic attraction of the weak acid anion by positively charged amino acid residues (39). The level of intricacy of the translocation mechanism increases from the AQPs via FNTs to the MCTs.

AQPs represent the least efficient weak acid facilitators and require strongly acidic conditions. Clusters of positively charged amino acids at the protein surface of, e.g. *Lactobacillus* AQPs attract l-lactate anions (34, 35) and locally enhance the probability of substrate protonation for passing the lipophilic channel (21). The FNTs are lookalikes of the AQPs (36). Despite unrelated protein sequences, they adopted the same channel-like fold, yet a positively charged lysine is positioned deep inside funnel-like vestibules on either side of the central channel section (2). A weak acid anion is attracted into the increasingly hydrophobic environment within a vestibule by the “dielectric slide” mechanism (37). At a certain point, the shifting substrate pK_a_ allows a proton to bind. The neutral acid passes a lipophilic constriction which additionally selects by size exclusion (38). The FNT layout enables proton-driven weak acid transport at physiological pH of similar efficiency as the MCTs. Yet, the FNTs exhibit electrogenic anion leakage at neutral pH (40). With the advent of the metazoa, the channel-like FNTs (41) were replaced by the alternating access MCTs (13). In the MCTs, two central steps of weak acid transport appear to be maintained, namely attraction of the acid anion and subsequent proton binding by the substrate. The additional employment of a proton transfer via the bound substrate to the switching amino acid Asp 309 is probably responsible for eliminating anion leakage altogether.

In conclusion, the transport mechanism of the MCTs supports the notion, that, throughout species, a hallmark of membrane protein-facilitated weak acid transport is the exploitation of the substrate-inherent physicochemical properties as a proton acceptor and/or transfer site for selectivity and activity in the physiological pH range.

## Materials and methods

### Plasmids, cloning, and mutagenesis

Human MCT1 in pDRT196 with N-terminal hemagglutinin- and C-terminal His_10_-tags was generated previously (17). Primers for site-directed mutagenesis are listed in Table S1. All constructs were sequenced.

#### Yeast transformation and culture

*S. cerevisiae* yeast lacking endogenous monocarboxylate transporters (W303-1A Δjen1 Δady2; MATa *leu2-3,112 trp1-1 can1-100 ura3-1 ade2-1 his3-11,15 jen1::kanMX4 ady2::hphMX4*) was kindly provided by M. Casal. Cells were transformed using the PEG/LiAc/ss-carrier DNA-method (42). Cells were grown at 29°C, 220 rpm in synthetic defined (SD) liquid media containing 2% (w/v) glucose, l-histidine (20 mg l^−1^), l-tryptophan (10 mg l^−1^), l-leucine (100 mg l^−1^), and adenine (25 mg l^−1^), lacking uracil for selection.

#### Cell disruption and western blotting

Here 40 ml cultures were harvested (4,000 *g*, 5 min, 4°C, OD_600_ of 1 ± 0.1), and washed with 50 ml water and 10 ml 25 mM TRIS, pH 7.5, 5 mM EDTA-buffer (TE). Pelleted cells were stored at −80°C. For disruption, cells were resuspended in 0.5 ml TE-buffer, repeatedly vortexed with an equal volume of acid-washed glass beads (Ø 0.5 mm; 15 intervals of 30 s and 1 min on ice), and cleared (1,000 *g*, 5 min, 4°C). Microsomal proteins were prepared by removal of cell debris (10,000 *g*, 5 min, 4°C), followed by ultracentrifugation (100,000 *g*, 40 min, 4°C). Membrane pellets were resuspended in 100 mM Na_2_HPO_4_, pH 8.0, 50 mM NaCl. Total protein was quantified using Bio-Rad Protein Assay (BioRad, Feldkirchen, Germany), separated by SDS-PAGE (12.5% acrylamide, peqGold III protein marker; VWR, Darmstadt, Germany), and blotted on PVDF (Hybond N, GE HealthCare, Solingen, Germany). Proteins were detected using monoclonal mouse-anti-His_5_ (Qiagen, Hilden, Germany), secondary goat-anti-mouse antibodies (Jackson ImmunoResearch, Cambridgeshire, United Kingdom), and Clarity ECL (BioRad, Feldkirchen, Germany) in a ChemoStar Touch cabinet (Intas, Göttingen, Germany).

### pH/pD-adjusted buffers used in ^14^C-labeled transport assays

Standard buffers were 50 mM/50 mM TRIS/HEPES (pH 8.3, 7.8, 7.5, 7.3, 7.0, 6.8, 6.3), TRIS/MES (pH 6.6, 5.8), and TRIS/citric acid (pH 5.3, 4.8, 4.3, 3.8, 3.3, 2.8). For determining isotope effects, phosphate/citric acid buffers were used (43). 0.2 M Na_2_HPO_4_ and 0.1 M citric acid stocks were prepared in light, H_2_O, and heavy water, D_2_O. The heavy water stocks were combined in different ratios for pD 6.8, 5.8, 4.8, and 3.8. pD was adjusted using a D_2_O-equilibrated and calibrated glass electrode (44). For light water buffers, the stocks were combined in the same ratios as above. Due to higher dissociation in light water, pH was adjusted using NaOH. This procedure assured equal concentrations of protons and deuterons at each pH/pD condition.

#### Radiolabeled l-lactate uptake assay

Uptake of radiolabeled l-lactate was assayed as previously (45). Briefly, yeast was harvested (4,000 *g*, 5 min, 4°C, OD_600_ of 1 ± 0.1), and washed with water. The cells were resuspended in pH/pD-adjusted buffer to an OD_600_ of 50 ± 5, and kept on ice for 30 min. For the assay, the cells were prewarmed to 19°C. Substrate uptake was initiated by adding ^14^C-spiked (0.04 µCi Hartmann Analytic, Braunschweig, Germany) l-lactate solution (1 mM final). Transport was stopped with 1 ml ice-cold water, and suction through GF/C glass microfiber filters (GE Healthcare, Solingen, Germany). The washed filters were placed in 3 ml of ROTISZINTeco plus (Carl Roth, Karlsruhe, Germany) overnight, and counted for 2 min (Packard TriCarb 2900 TR, Perkin Elmer). For Michaelis–Menten kinetics, substrate and radiolabel were increased up to 50 mM and 0.14 µCi. l-Lactamide competition was assayed at 4 mM l-lactate plus 4 mM or 40 mM l-lactamide. Batches in light and heavy water were prepared from the same yeast culture. Internalized l-lactate was normalized to 1 mg of yeast, and background radiolabel on non-expressing cells was subtracted. Uptake rates were determined from the initial linear phase of the curves (usually 2 min, 8 min for slow transport). Michaelis–Menten parameters were calculated from exponential curve fittings (K_m_ = ln(0.5)/rate constant; v_max_ = limit of the curve). Measurements were done in duplicates with two to six biological replicates.

#### Monitoring of changes in yeast protoplast volume by stopped-flow light scattering

The assay was adapted from previous work (21, 23). Here, 50 ml transformed yeast were harvested (2,000 *g*, 5 min, 4°C, OD_600_ of 1 ± 0.1), washed and resuspended (2 ml) in 50 mM MOPS buffer, pH 7.2, with 0.2% of freshly added 2-mercaptoethanol. For protoplastation, zymolyase 20 T (400 U per gram of yeast cells), 100 mg of bovine serum albumin (fraction V), and 1.2 M sucrose (all from Carl Roth, Karlsruhe, Germany) were added and incubated for 1 h at 30°C, 140 rpm. Protoplasts were collected (2,000 *g*, 5 min, 4°C), washed once in 5 ml of 20 mM MOPS buffer, pH 7.2, plus 1.2 M sucrose, 50 mM NaCl and 5 mM CaCl_2_, and diluted in this buffer to an OD_600_ of 2. For the assay, 75 µl of protoplast suspension were mixed using a stopped-flow device (SFM-200, BioLogic, Seyssinet-Pariset, France) with an equal volume of buffer supplemented with 600 mM l-lactate, chloroacetate, glycerol, l-lactamide, or l-proline (300 mM inward gradient). For an assay pH of 5.1 ± 0.3 the protoplast suspension was mixed with a pH 4.5 ± 0.3 buffer. Further parameters were: 20°C, dead time 2.7 ms, flow rate 8 ml s^−1^. The intensity of 90° light scattering (λ = 524 nm) was monitored. Nine traces were averaged, normalized, and done in three biological replicates. Substrate uptake velocity was derived from by linear fits (20–90 s range) of background-subtracted traces. Significance of was tested using one-tailed one-sample t-tests. Asterisks indicate *P*-values <0.05.

## Protein structure visualization and solvation simulation

MCT1 protein structure displays were done with the Chimera software (46). Solvation of the MCT1 binding pocket in the outward open conformation was simulated by placing the PDB structure file 6LZ0 (12) in an SPC/Fw water shell with an extent of 5 Å using the AMBER tools (47) from within the Chimera software.

## Supplementary Material

pgad007_Supplementary_Data

## Data Availability

All data generated or analyzed during this study are included in this published article (and its Supplementary Material files).

## References

[1] Manallack DT , et al 2013. The acid/base profile of the human metabolome and natural products. Mol Inf. 32:505–515. 10.1002/minf.201200167 27481668

[2] Wiechert M , Beitz E. 2017. Mechanism of formate-nitrite transporters by dielectric shift of substrate acidity. EMBO J. 36:949–958. 28250043 10.15252/embj.201695776 PMC5376963

[3] Bröer S , et al 1998. Characterization of the monocarboxylate transporter 1 expressed in *Xenopus laevis* oocytes by changes in cytosolic pH. Biochem J. 333:167–174. 9639576 10.1042/bj3330167 PMC1219569

[4] Bröer S , et al 1999. Characterization of the high-affinity monocarboxylate transporter MCT2 in Xenopus laevis oocytes. Biochem J. 341:529–535. 10417314 10.1042/0264-6021:3410529 PMC1220388

[5] Yoon H , Fanelli A, Grollman EF, Philp NJ. 1997. Identification of a unique monocarboxylate transporter (MCT3) in retinal pigment epithelium. Biochem Biophys Res Commun. 234:90–94. 9168967 10.1006/bbrc.1997.6588

[6] Manning Fox JE , Meredith D, Halestrap AP. 2000. Characterisation of human monocarboxylate transporter 4 substantiates its role in lactic acid efflux from skeletal muscle. J Physiol. 529:285–293. 11101640 10.1111/j.1469-7793.2000.00285.x PMC2270204

[7] Nakajima EC , Van Houten B. 2013. Metabolic symbiosis in cancer: refocusing the Warburg lens. Mol Carcinog. 52:329–337. 22228080 10.1002/mc.21863 PMC9972501

[8] Sonveaux P , et al 2008. Targeting lactate-fueled respiration selectively kills hypoxic tumor cells in mice. J Clin Invest. 118:3930–3942. 19033663 10.1172/JCI36843 PMC2582933

[9] Le Floch R , et al 2001. CD147 Subunit of lactate/H^+^ symporters MCT1 and hypoxia-inducible MCT4 is critical for energetics and growth of glycolytic tumors. Proc Natl Acad Sci U S A. 108:16663–16668. 10.1073/pnas.1106123108 PMC3189052 21930917

[10] Bosshart PD , Kalbermatter D, Bonetti S, Fotiadis D. 2019. Mechanistic basis of l-lactate transport in the SLC16 solute carrier family. Nat Commun. 10:2649. 31201333 10.1038/s41467-019-10566-6 PMC6573034

[11] Zhang B , et al 2020. Cooperative transport mechanism of human monocarboxylate transporter 2. Nat Commun. 11:2429. 32415067 10.1038/s41467-020-16334-1 PMC7228944

[12] Wang N , et al 2021. Structural basis of human monocarboxylate transporter 1 inhibition by anti-cancer drug candidates. Cell. 184:370–383.e13. 33333023 10.1016/j.cell.2020.11.043

[13] Liu Q , Dou S, Wang G, Li Z, Feng Y. 2008. Evolution and functional divergence of monocarboxylate transporter genes in vertebrates. Gene. 423:14–22. 18674605 10.1016/j.gene.2008.07.003

[14] Manoharan C , Wilson MC, Sessions RB, Halestrap AP. 2006. The role of charged residues in the transmembrane helices of monocarboxylate transporter 1 and its ancillary protein basigin in determining plasma membrane expression and catalytic activity. Mol Membr Biol. 23:486–498. 17127621 10.1080/09687860600841967 PMC2409183

[15] Wilson MC , Meredith D, Bunnun C, Sessions RB, Halestrap AP. 2009. Studies on the DIDS-binding site of monocarboxylate transporter 1 suggest a homology model of the open conformation and a plausible translocation cycle. J Biol Chem. 284:20011–20021. 19473976 10.1074/jbc.M109.014217 PMC2740427

[16] Rahman B , Schneider HP, Bröer A, Deitmer JW, Bröer S. 1999. Helix 8 and helix 10 are involved in substrate recognition in the rat monocarboxylate transporter MCT1. Biochemistry. 38:11577–11584. 10471310 10.1021/bi990973f

[17] Köpnick A-L , Jansen A, Geistlinger K, Epalle NH, Beitz E. 2021. Basigin drives intracellular accumulation of l-lactate by harvesting protons and substrate anions. PLoS One. 16:e0249110. 33770122 10.1371/journal.pone.0249110 PMC7996999

[18] Köpnick A-L , Geistlinger K, Beitz E. 2021. Cysteine 159 delineates a hinge region of the alternating access monocarboxylate transporter 1 and is targeted by cysteine-modifying inhibitors. FEBS J. 288:6052–6062. 33999492 10.1111/febs.16024

[19] Geistlinger K , Schmidt JDR, Beitz E. 2022. Lactic acid permeability of aquaporin-9 enables cytoplasmic lactate accumulation via an ion trap. Life. 12:120. 35054513 10.3390/life12010120 PMC8779662

[20] Yamaguchi A , et al 2020. Extracellular lysine 38 plays a crucial role in pH-dependent transport via human monocarboxylate transporter 1. Biochim Biophys Acta Biomembr. 1862:183068. 31593685 10.1016/j.bbamem.2019.183068

[21] Rothert M , Rönfeldt D, Beitz E. 2017. Electrostatic attraction of weak monoacid anions increases probability for protonation and passage through aquaporins. J Biol Chem. 292:9358–9364. 28360107 10.1074/jbc.M117.782516 PMC5454115

[22] De Bruijne AW , Vreeburg H, Van Steveninck J. 1983. Kinetic analysis of l-lactate transport in human erythrocytes via the monocarboxylate-specific carrier system. Biochim Biophys Acta. 732:562–568. 6871216 10.1016/0005-2736(83)90232-8

[23] Helmstetter F , Arnold P, Höger B, Petersen LM, Beitz E. 2019. Formate–nitrite transporters carrying nonprotonatable amide amino acids instead of a central histidine maintain pH-dependent transport. J Biol Chem. 294:623–631. 30455351 10.1074/jbc.RA118.006340 PMC6333897

[24] Carpenter L , Halestrap AP. 1994. The kinetics, substrate and inhibitor specificity of the lactate transporter of Ehrlich-Lettre tumour cells studied with the intracellular pH indicator BCECF. Biochem J. 304:751–760. 7818477 10.1042/bj3040751 PMC1137398

[25] Sasaki S , Futagi Y, Kobayashi M, Ogura J, Iseki K. 2015. Functional characterization of 5-oxoproline transport via SLC16A1/MCT1. J Biol Chem. 290:2303–2311. 25371203 10.1074/jbc.M114.581892 PMC4303682

[26] Bell RP , Kuhn AT. 1963. Dissociation constants of some acids in deuterium oxide. Trans Faraday Soc. 59:1789–1793.

[27] Lewis GN , Doody TC. 1933. The mobility of ions in H_2_H_2_O. J Am Chem Soc. 55:3504–3506.

[28] DeCoursey TE , Cherny VV. 1997. Deuterium isotope effects on permeation and gating of proton channels in rat alveolar epithelium. J Gen Physiol. 109:415–434. 9101402 10.1085/jgp.109.4.415 PMC2219434

[29] Vasilescu V , Mărgineanu D-G, Katona E. 1977. Heavy water intake in tissues. II.H_2_O-D_2_O exchange in the myelinated nerve of the frog. Experientia. 33:192–194. 844550 10.1007/BF02124060

[30] Naftalin RJ , Rist RJ. 1991. 3-O-methyl-d-glucose Transport in rat red cells: effects of heavy water. Biochim Biophys Acta. 1064:37–48. 1851040 10.1016/0005-2736(91)90409-2

[31] Fernandez PL , Murkin AS. 2020. Inverse solvent isotope effects in enzyme-catalyzed reactions. Molecules. 25:1933. 32326332 10.3390/molecules25081933 PMC7221790

[32] Ruprecht JJ , et al 2019. The molecular mechanism of transport by the mitochondrial ADP/ATP carrier. Cell. 176:435–447.e15. 30611538 10.1016/j.cell.2018.11.025 PMC6349463

[33] Noor SL , et al 2018. A surface proton antenna in carbonic anhydrase II supports lactate transport in cancer cells. Elife. 7:e35176. 29809145 10.7554/eLife.35176 PMC5986270

[34] Bienert GP , Desguin B, Chaumont F, Hols P. 2013. Channel-mediated lactic acid transport: a novel function for aquaglyceroporins in bacteria. Biochem J. 454:559–570. 23799297 10.1042/BJ20130388

[35] Schmidt JDR , Walloch P, Höger B, Beitz E. 2021. Aquaporins with lactate/lactic acid permeability at physiological pH conditions. Biochimie. 188:7–11. 33577940 10.1016/j.biochi.2021.01.018

[36] Wang Y , et al 2009. Structure of the formate transporter FocA reveals a pentameric aquaporin-like channel. Nature. 462:467–472. 19940917 10.1038/nature08610

[37] Wiechert M , Beitz E. 2017. Formate-nitrite transporters: monoacids ride the dielectric slide. Channels. 11:365–367. 28494190 10.1080/19336950.2017.1329999 PMC5626355

[38] Wiechert M , Erler H, Golldack A, Beitz E. 2017. A widened substrate selectivity filter of eukaryotic formate-nitrite transporters enables high-level lactate conductance. FEBS J. 284:2663–2673. 28544379 10.1111/febs.14117

[39] Schmidt JDR , Beitz E. 2022. Mutational widening of constrictions in a formate-nitrite/H^+^ transporter enables aquaporin-like water permeability and proton conductance. J Biol Chem. 298:101513. 34929166 10.1016/j.jbc.2021.101513 PMC8749060

[40] Lü W , et al 2012. The formate channel FocA exports the products of mixed-acid fermentation. Proc Natl Acad Sci U S A. 109:13254–13259. 22847446 10.1073/pnas.1204201109 PMC3421167

[41] Mukherjee M , Vajpai M, Sankararamakrishnan R. 2017. Anion-selective formate/nitrite transporters: taxonomic distribution, phylogenetic analysis and subfamily-specific conservation pattern in prokaryotes. BMC Genomics. 18:560. 28738779 10.1186/s12864-017-3947-4 PMC5525234

[42] Gietz RD . 2014. Yeast transformation by the LiAc/SS carrier DNA/PEG method. Methods Mol Biol. 1163:33–44. 24841298 10.1007/978-1-4939-0799-1_4

[43] McIlvaine TC . 1921. A buffer solution for colorimetric comparison. J Biol Chem. 49:183–186.

[44] Bates RG . 1968. Standardization of acidity measurements extension of the pH concept to mixed solvents and heavy water. Anal Chem. 40:28–38.

[45] Wu B , et al 2015. Identity of a *Plasmodium* lactate/H^+^ symporter structurally unrelated to human transporters. Nat Commun. 6:6284. 25669138 10.1038/ncomms7284

[46] Pettersen EF , et al 2004. UCSF Chimera a visualization system for exploratory research and analysis. J Comput Chem. 25:1605–1612. 15264254 10.1002/jcc.20084

[47] Case DA , et al 2022. Amber. San Francisco: University of California.

