# Supplementary material for: Human monocarboxylate transporters accept and relay protons via the bound substrate for selectivity and activity at physiological pH
Source: PNAS Nexus. 2023 Jan 18;2(2):pgad007. doi: 10.1093/pnasnexus/pgad007 (PMC9982067; doi:10.1093/pnasnexus/pgad007)
Supplement: pgad007_Supplementary_Data [file pgad007_supplementary_data.pdf]

PNAS nexus

Supplementary Information for

Human monocarboxylate transporters accept and relay protons via the bound substrate for selectivity and activity at physiological pH

Katharina Geistlinger, Jana D.R. Schmidt, Eric Beitz

Eric Beitz
Email: ebeitz@pharmazie.uni-kiel.de

This PDF file includes:

Figures S1 to S5
Table S1

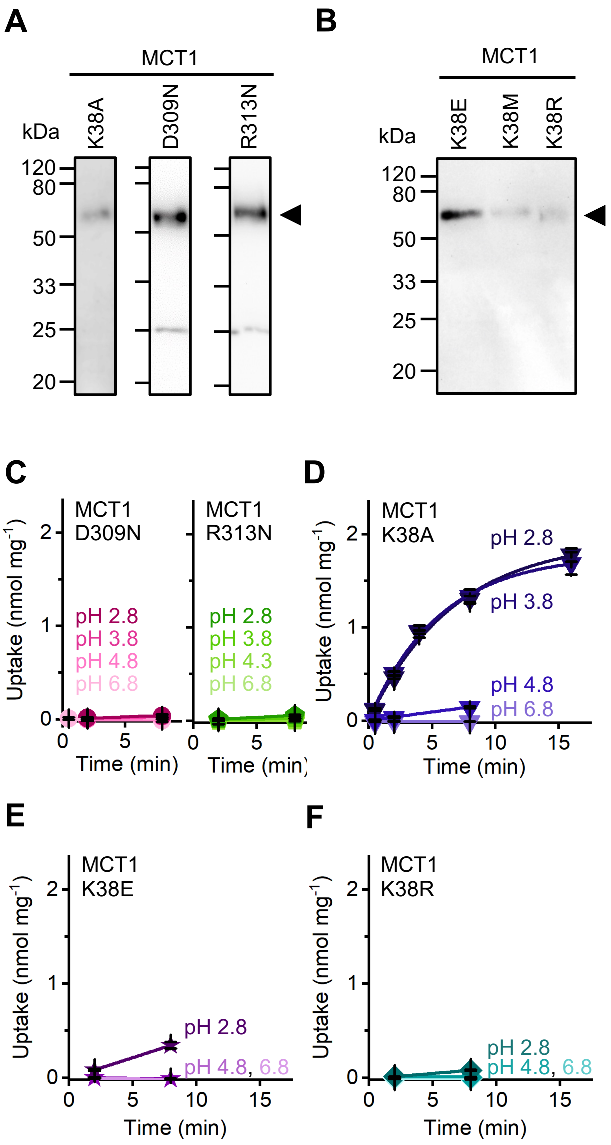

Fig. S1. Transport functionality of MCT1 Lys 38 mutants MCT1 K38A, MCT1 K38E, and MCT1 K38R. **A** and **B**. Western blots showing expression of MCT1 point mutants in *S. cerevisiae* Δ*jen1* Δ*ady2* yeast detected via C-terminal His_{10}-tags at the expected size of 58 kDa (arrow head). **C-F**. L-Lactate uptake over time into yeast via MCT1 mutants D309N, R313N, K38A, K38E, or K38R at a 1 mM inward substrate gradient and different buffer pH conditions. Error bars denote S.E.M. (n = 3; except for K38A at pH 6.8 and 4.8: n = 2; non-functional D309N and R313N: n = 1-2; all done in technical duplicates).

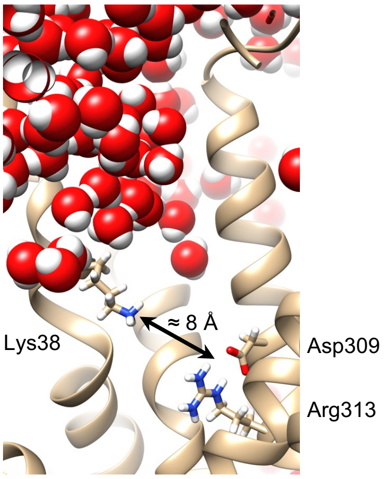

Fig. S2. Simulation of the accessibility of the MCT1 substrate binding pocket by bulk water.

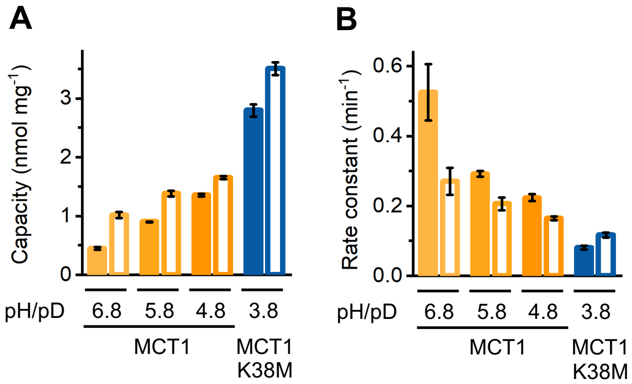

Fig. S3. Heavy water effect on L-lactate transport via MCT1 wildtype and K38M. **A**. Capacities of transport into yeast in light (closed bars) and heavy water (open bars). **B**. Rate constants of MCT transport in light (closed bars) and heavy water (open bars). Data were derived from the curves in Fig. 3D of the main paper.

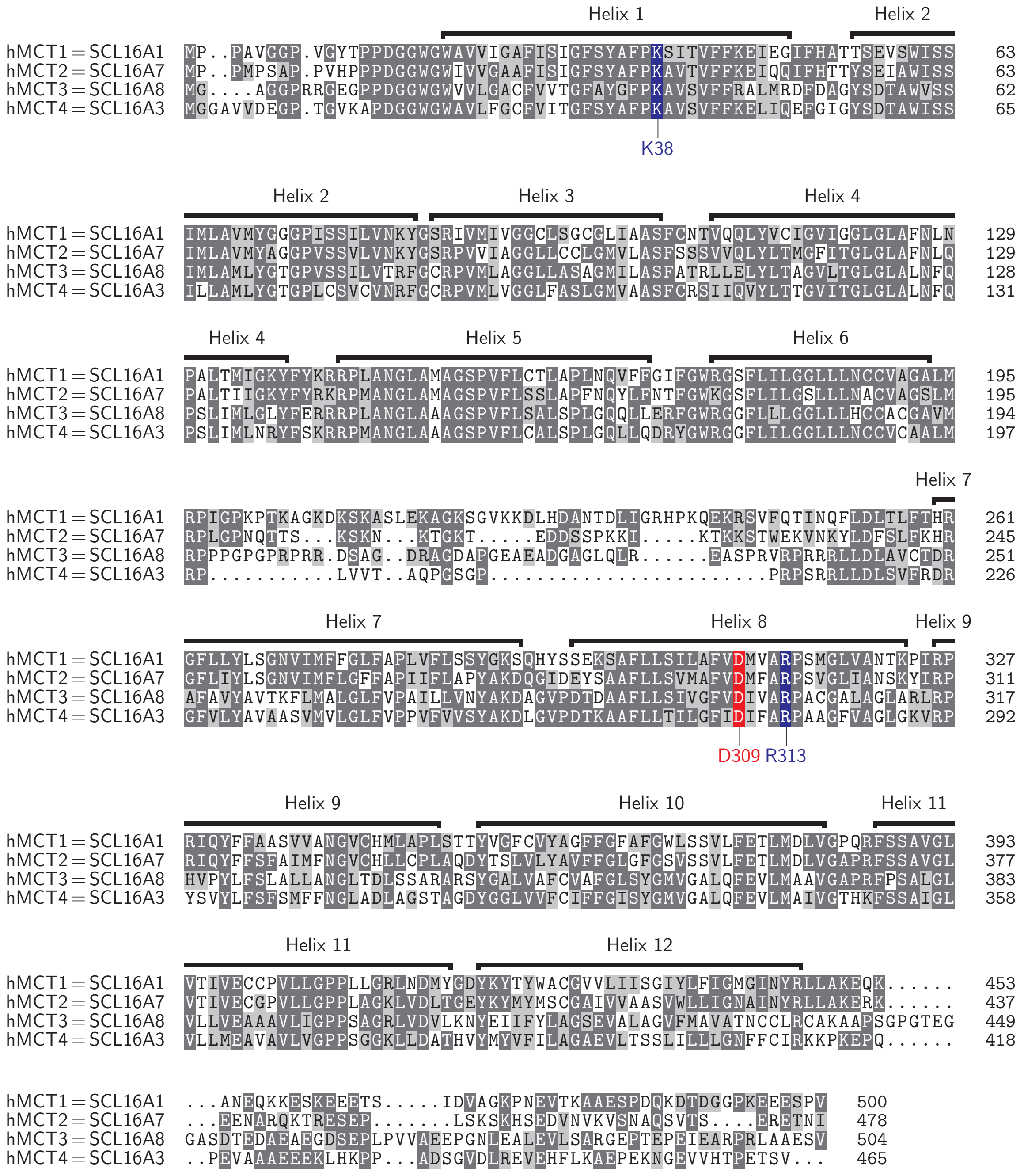

Fig. S4. Sequence alignment of human MCT1-4. The three charged residues of the L-lactate binding site (K38, D309, R313) are labeled below.

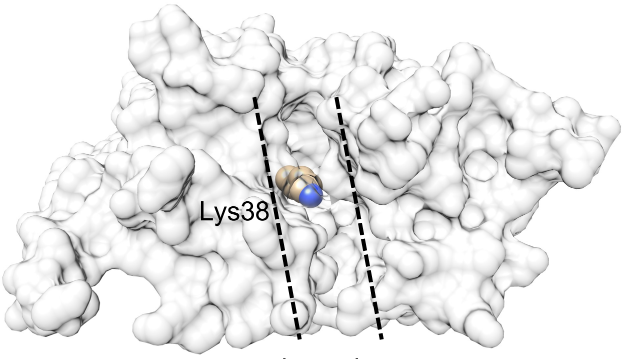

Fig. S5. Inward open conformation of MCT1 D309N. Shown is the view on Lys 38 from the cytoplasmic side (PDB# 7DA5). The ε-amine appears to be accessible to a substrate anion from the intracellular side.

Table S1. MCT1 mutation primers (non-complementary nucleotides in lower-case).

K38A, sense	5′-TCT TAT GCA TTT CCC gcg TCA ATT ACT GTC-3′
K38E, sense	5′-TCT TAT GCA TTT CCC gAA TCA ATT ACT GTC-3′
K38M, sense	5′-TCT TAT GCA TTT CCC Atg TCA ATT ACT GTC-3′
K38R, sense	5′-TCT TAT GCA TTT CCC AgA TCA ATT ACT GTC-3′
K38X, antisense	5′-GGG AAA TGC ATA AGA GAA GCC GAT GG-3′
D309N, sense	5′-ATT CTG GCT TTT GTT aAt ATG GTA GCC CG-3′
D309N, antisense	5′-AAC AAA AGC CAG AAT GGA AAG AAG GAA GGC-3′
R313N, sense	5′-GTT GAC ATG GTA GCC aat CCA TCT ATG GGA CTT-3′
R313N, antisense	5′-GGC TAC CAT GTC AAC AAA AGC CAG AAT GGA-3′.
